# Supplementary material for: Genome-wide mega-analysis identifies 16 loci and highlights diverse biological mechanisms in the common epilepsies
Source: Nat Commun. 2018 Dec 10;9:5269. doi: 10.1038/s41467-018-07524-z (PMC6288131; doi:10.1038/s41467-018-07524-z)
Supplement: Supplementary file 1 — Supplementary Information [file 41467_2018_7524_MOESM1_ESM.pdf]

Genome-wide mega-analysis identifies 16 loci and highlights diverse biological mechanisms in the common epilepsies

The International League Against Epilepsy Consortium on Complex Epilepsies

Supplementary Tables ____ 2

Supplementary Table 1: Overview of number of epilepsy cases stratified by phenotype and ethnicity. ____ 2
Supplementary Table 2: Validation of the 6 biological prioritization criteria ____ 3
Supplementary Table 3: Transcriptome-wide association analysis (TWAS) ____ 4
Supplementary Table 4: Examples of significant drug repurposing candidates ____ 5
Supplementary Table 5: The genetic correlations of epilepsy with other phenotypes ____ 6
Supplementary Table 6: Details of participating cohorts ____ 8
Supplementary Table 7: Estimation of inflation factor and the LD-score regression intercept ____ 8
Supplementary Table 8: List of selected monogenic epilepsy genes ____ 8
Supplementary Table 9: brain-coX in silico gene co-expression prioritization ____ 10
Supplementary Table 10: List of genes that are targets of currently used anti-epileptic drugs ____ 11
Supplementary Table 11: SNP-based heritabilities ____ 11
Supplementary Table 12: Comparative SNP-based heritability estimates ____ 12

Supplementary Figures ____ 13

Supplementary Figure 1: Principal component plots ____ 13
Supplementary Figure 2: Quantile-quantile (Q-Q) plots. Q-Q plots of meta-analyses ____ 14
Supplementary Figure 3: Conditional analysis of 2q24.3 ____ 15
Supplementary Figure 4: Manhattan plots for the 7 epilepsy subtypes ____ 16
Supplementary Figure 5: Q-Q plots of the 7 epilepsy subtypes ____ 17
Supplementary Figure 6: Forest plot of the lead SNPs ____ 18
Supplementary Figure 7: Locuszoom plots for all genome-wide significant loci. Genome-wide significant loci _ 21
Supplementary Figure 8: GWAS results of genetic generalized epilepsy conditioned on expression of PNPO __ 21
Supplementary Figure 9: Gene co-expression matrix produced by brain-coX ____ 22
Supplementary Figure 10: Heritability enrichment of 6 different chromatin markers in 88 tissues ____ 23
Supplementary Figure 11: Heritability enrichment of genes expressed in 53 tissues ____ 24
Supplementary Figure 12: MAGMA gene expression enrichment analysis ____ 25
Supplementary Figure 13: Differentially expressed genes across the lifespan ____ 26
Supplementary Figure 14: Results of MTAG analysis ____ 26
Supplementary Figure 15: Power curves for GWAS ____ 27

Supplementary References ____ 28

Supplementary Tables

Phenotype	Epilepsy sub-phenotypes	n	CAU	ASI	AFR
Generalized	Generalized Epilepsy, not otherwise specified, with spike and wave EEG	1,297	1,258	0	33
	Childhood Absence Epilepsy (CAE)	793	778	0	15
	Juvenile Absence Epilepsy (JAE)	415	415	0	0
	Juvenile Myoclonic Epilepsy (JME)	1,181	1,177	0	10
	GTCS alone, with spike and wave EEG	228	225	0	3
	**Subtotal**	**3,769***	**3,708***	**0**	**61**
Focal	Focal Epilepsy, not otherwise specified	3,082	2,975	49	58
	Focal Epilepsy, documented lesion negative	2,716	2,660	31	25
	Focal Epilepsy, documented hippocampal sclerosis (HS)	803	709	91	3
	Focal Epilepsy, documented lesion other than HS	3,070	2,751	319	0
	**Subtotal**	**9,671**	**9,095**	**490**	**86**
Unclassified	Epilepsy, not otherwise specified	1,772	1,731	41	0
**Cases**		**15,212**	**14,534**	**531**	**147**
**Controls**		**29,677**	**24,218**	**2,875**	**2,584**
**Total subjects**		**44,889**	**38,752**	**3,406**	**2,731**

Supplementary Table 1: Overview of number of epilepsy cases stratified by phenotype and ethnicity. *After quality controls we had 15,212 cases and 29,677 controls. Subjects were classified into focal or genetic generalized epilepsy (and their respective syndromes), or unclassified epilepsy. CAU: Caucasian, ASI: Asian, AFR: African. *145 subjects with dual CAE/JAE diagnosis were included in both the analyses of CAE and JME respectively.*

	TWAS	eQTL	Brain exp	Missense	PPI	KO mouse	Total score
Correlation coef	0.07	0.08	0.33	0.13	0.14	0.26	**0.42**
Sensitivity	0.17	0.17	1.00	0.17	0.50	0.83	**0.83**
Specificity	0.93	0.94	0.74	0.96	0.79	0.75	**0.89**
Postive predictive value	0.09	0.10	0.14	0.17	0.09	0.13	**0.24**
Negative predictive value	0.96	0.96	1.00	0.96	0.97	0.99	**0.99**

0

1

Supplementary Table 2: Validation of the 6 biological prioritization criteria (Figure 2) to predict established monogenic epilepsy genes. A list of 102 established monogenic epilepsy genes (see Supplementary Table 8) was used to calculate the correlation, sensitivity, specificity, positive predictive value and negative predictive value of the prioritization criteria in predicting established epilepsy genes out of the 146 genes that were mapped to genome-wide significant loci (Supplementary Date file 1). The total score is the sum of criteria being met per gene (range 0-6). Correlation coefficients are calculated as Pearson correlations. The "total score" column represents our approach of selecting the gene(s) with the highest score within each locus as prioritized epilepsy genes.

Phenotype	Gene ID	HSQ	BEST.GWAS.ID	EQTL.ID	MODEL	MODELCV.R2	MODELCV.PV	TWAS.Z	TWAS.P
All epilepsy	HEATR3	0.1472	rs4638568	rs7186889	lasso	0.19531	2.97E-23	1.0508	0.293351
	ADCY7	0.1011	rs4638568	rs1362622	enet	0.05077	7.76E-07	-0.21526	0.829565
	VRK2	0.0677	rs4671319	rs11125739	bslmm	0.015528	4.59E-03	0.099582	0.920676
	FANCL	0.0611	rs4671319	rs11682175	blup	0.029378	1.48E-04	3.443959	0.000573
	COBLL1	0.0496	rs10497257	rs3769876	lasso	0.026154	3.27E-04	0.22521	0.821816
	GALNT3	0.075	rs6731869	rs6717367	lasso	0.069706	7.21E-09	-3.06498	0.002177
	SCN9A	0.0974	rs6731869	rs13034090	bslmm	0.075715	1.62E-09	1.141746	0.25356
	SCN7A	0.1072	rs6731869	rs3748895	bslmm	0.047216	1.86E-06	-1.498476	0.13401
Focal epilepsy	COBLL1	0.0496	rs4667729	rs3769876	lasso	0.026154	3.27E-04	-0.578968	0.562611
	GALNT3	0.075	rs6731869	rs6717367	lasso	0.069706	7.21E-09	-1.950729	0.051089
	SCN9A	0.0974	rs6731869	rs13034090	bslmm	0.075715	1.62E-09	1.218455	0.223051
	SCN7A	0.1072	rs6731869	rs3748895	bslmm	0.047216	1.86E-06	-0.652246	0.514242
Genetic generalized epilepsy	TBKBP1	0.0316	rs886444	rs9901869	blup	0.00604	5.38E-02	2.6009	9.30E-03
	MRPL10	0.3881	rs886444	rs3809869	lasso	0.44771	3.30E-60	-0.6047	5.45E-01
	SCRN2	0.3397	rs886444	rs17856536	lasso	0.23108	9.89E-28	-0.1407	8.88E-01
	PNPO	0.1487	rs886444	rs12602010	bslmm	0.09123	3.29E-11	4.8563	1.20E-06
	CDK5RAP3	0.1518	rs886444	rs2240119	bslmm	0.13552	3.58E-16	-2.0837	3.72E-02
	SNX11	0.0383	rs886444	rs11079808	lasso	0.02549	3.85E-04	-0.177	8.60E-01
	UBXN2A	0.2198	rs1862902	rs12616678	enet	0.235152	2.97E-28	-0.25073	8.02E-01
	TP53I3	0.1931	rs1862902	rs3731620	lasso	0.125388	5.10E-15	1.53797	1.24E-01
	VRK2	0.0677	rs1402398	rs11125739	bslmm	0.015528	4.59E-03	0.62332	5.33E-01
	FANCL	0.0611	rs1402398	rs11682175	blup	0.029378	1.48E-04	4.72457	2.31E-06
	COBLL1	0.0496	rs6729185	rs3769876	lasso	0.026154	3.27E-04	1.19094	2.34E-01
	GALNT3	0.075	rs11890028	rs6717367	lasso	0.069706	7.21E-09	-2.76521	5.69E-03
	SCN9A	0.0974	rs11890028	rs13034090	bslmm	0.075715	1.62E-09	0.83388	4.04E-01
	SCN7A	0.1072	rs11890028	rs3748895	bslmm	0.047216	1.86E-06	-0.74225	4.58E-01
	HIBCH	0.4269	rs7590384	rs16832572	lasso	0.336432	3.28E-42	3.13528	1.72E-03
	INPP1	0.1398	rs7590384	rs1882891	lasso	0.133908	5.47E-16	-1.85941	6.30E-02
	MFSD6	0.0637	rs7590384	rs10931458	enet	0.045795	2.63E-06	2.1394	3.24E-02
	NAB1	0.0623	rs7590384	rs2293765	lasso	0.043763	4.33E-06	1.80349	7.13E-02
	STAT4	0.0664	rs7590384	rs6738544	lasso	0.040593	9.42E-06	0.54052	5.89E-01

	GNPDA2	0.1597	rs10155504	rs12499960	enet	0.116163	5.61E-14	-0.60636	0.544274
	GABRA2	0.0517	rs10517160	rs9291283	bslmm	0.021086	1.14E-03	-3.7157	0.000203
	TRIM36	0.0673	rs4596374	rs10059320	bslmm	1.50E-02	5.30E-03	0.2226	0.823843
	PGGT1B	0.1173	rs4596374	rs9326953	enet	6.83E-02	1.03E-08	-0.82271	0.410672
	CCDC112	0.0382	rs4596374	rs6870296	blup	7.60E-03	3.54E-02	2.91427	0.003565
	PTPRK	0.0797	rs6905941	rs1508439	bslmm	0.048057	1.51E-06	-3.66355	0.000249
JME	RNF40	0.1351	rs1046276	rs7187359	enet	0.14652	1.93E-17	3.5323	4.12E-04
	SETD1A	0.0356	rs1046276	rs11150599	bslmm	0.01623	3.85E-03	5.6491	1.61E-08
	HSD3B7	0.0471	rs1046276	rs7197717	lasso	0.07076	5.54E-09	5.1649	2.41E-07
	ZNF646	0.0413	rs1046276	rs749671	lasso	0.04362	4.48E-06	4.9236	8.50E-07
	VKORC1	0.029	rs1046276	rs4889490	lasso	0.03383	4.94E-05	-3.342	8.32E-04
	KAT8	0.0641	rs1046276	rs749767	lasso	0.07213	3.95E-09	3.302	9.60E-04
	PRSS36	0.1446	rs1046276	rs889555	enet	0.11267	1.38E-13	4.5402	5.62E-06
	TRIM72	0.0815	rs1046276	rs4889640	enet	0.04477	3.38E-06	-2.7042	6.85E-03
	ITGAX	0.0475	rs1046276	rs8052139	enet	0.0536	3.87E-07	-3.3345	8.54E-04
CAE	VRK2	0.0677	rs12185644	rs11125739	bslmm	0.015528	4.59E-03	0.91249	0.36151
	FANCL	0.0611	rs12185644	rs11682175	blup	0.029378	1.48E-04	3.47011	0.00052
	GTDC1	0.1455	rs1346343	rs10206546	lasso	0.125727	4.67E-15	0.432	0.66574
Focal HS	PLCH1	0.3139	rs13315001	rs13100169	lasso	0.23371	4.54E-28	1.11632	0.26429
	SLC33A1	0.1634	rs13315001	rs382534	bslmm	0.15404	2.58E-18	0.15728	0.87503
	KCNAB1	0.062	rs13315001	rs1027915	lasso	0.02701	2.65E-04	0.96785	0.33312
	FAM184A	0.2672	rs9489521	rs4945630	bslmm	0.210874	3.54E-25	-0.23076	0.8175

Supplementary Table 3: Transcriptome-wide association analysis (TWAS) to assess the association between epilepsy phenotypes with gene-expression, performed using RNA-sequencing data of dorsolateral prefrontal cortex tissue from the CommonMind Consortium. The TWAS was limited to the 146 genes that were mapped to genome-wide significant loci in their respective phenotypes. Gene-expression weights to calculate the TWAS statistic were available for 53 of these 146 genes. Significant TWAS correlations after Bonferroni correction (p<0.05/53=0.00094) are highlighted in red.

Name	Cosine distance	Indications	First licensed (year)	Number of studies	PMIDs	Number of unique models	Models
Pimozide	-0.61440782	Schizophrenia	1984	3	2272645, 6141554, 7875556	4	PTZ, Electro, AUD, Other
Progesterone	-0.612515285	Infertility; premenstrual symptoms; post-natal depression; recurrent miscarriage	1976	17	1356068, 9657647, 9918575, 10725616, 11166716, 12009770, 12821382, 14636318, 14982969, 15571511, 15820338, 16085296, 16784731, 19840816, 19907717, 20804775, 22835430	5	KA, PTZ, Electro, Kin, Other
Clonidine	-0.584639091	Migraine; Tourette syndrome; hypertension; menopausal flushing; sedation	1974	21	1486503, 1847879, 2508150, 2826214, 2848694, 2876688, 2885413, 2995952, 6104728, 6306699, 6504961, 6843298, 7160794, 7305545, 7938564, 10211593, 10372568, 17214976, 23756131, 24139618, 25765931	7	KA, Pilo, PTZ, Electro, AUD, Kin, Other
Fluvoxamine	-0.565953681	Depression; obsessive-compulsive disorder	1994	1	25590967	1	PTZ
Losartan	-0.511120986	Hypertension; heart failure; diabetic nephropathy	1995	7	8923498, 10544388, 20533906, 21797110, 24146309, 24659129, 25456349	3	KA, PTZ, Other
Amiodarone	-0.509678023	Arrhythmia	1985	1	19353751	2	PTZ, Other
Sertraline	-0.504973971	Depression; obsessive-compulsive disorder;	1991	2	7582562, 8538363	2	AUD, Other

		panic disorder; post-traumatic stress disorder; social anxiety disorder					
Haloperidol	-0.501865349	Schizophrenia	1967	1	7875556	1	Other
Chloroquine	-0.481150292	Malaria; rheumatoid arthritis; lupus erythematosus	1949	2	7816747, 26655695	1	PTZ
Rofecoxib	-0.477826512	Osteoarthritis; pain; dysmenorrhoea	1999	7	16671960, 16844276, 17139192, 17139446, 18054463, 18340407, 19907717	2	KA, PTZ
coFlutamide	-0.469883681	Prostate cancer	1989	2	10446327, 12752460	3	PTZ, Electro, Other
Promethazine	-0.469157198	Nausea; vomiting; vertigo; labyrinthine disorders; motion sickness; allergy; urticaria; sedation	1951	2	1874553, 2905589	1	Electro
Tetracycline	-0.466342012	Acne vulgaris; rosacea	1953	1	22579030	1	Electro
Raloxifene	-0.458846622	Postmenopausal osteoporosis	1997	1	25218046	1	KA
Modafinil	-0.457880158	Narcolepsy	1998	2	17681557, 25869564	2	PTZ, Electro
Diazoxide	-0.457875065	Hypoglycaemia	1973	2	15488295, 19728004	3	Pilo, Kin, Other
Amlodipine	-0.451374469	Hypertension; angina	1992	4	1698518, 11218825, 11526962, 20593148	3	Pilo, PTZ, Electro
Sildenafil	-0.447401021	Pulmonary hypertension; erectile dysfunction	1998	3	20067503, 20508294, 23994662	2	Electro, Kin
Verapamil	-0.441081591	Angina; hypertension; cluster headache; supraventricular arrhythmia	1981	12	1946038, 2279078, 2411508, 2450733, 2744396, 3393267, 3784769, 7681002, 8152336, 8156971, 8707372, 11218825	6	KA, Pilo, PTZ, AUD, Kin, Other
Riluzole	-0.438649286	Amyotrophic lateral sclerosis	1995	4	11070180, 11463511, 12457877, 20015615	4	KA, Pilo, Kin, Other
Nicardipine	-0.43461414	Angina; hypertension	1988	5	7681002, 8152336, 8872866, 10608279, 11742591	4	KA, PTZ, Kin, Other
Dexamethasone	-0.431661129	Inflammatory and allergic disorders; congenital adrenal hyperplasia; cerebral oedema; croup; rheumatic disease	1958	3	16445912, 21403895, 21464890	3	KA, Pilo, Kin
Bupropion	-0.429076912	Depression; smoking cessation	1985	3	15363958, 21962757, 23770308	3	KA, PTZ, Electro
Doxepin	-0.420870706	Depression; pruritus	1969	2	1456842, 19443935	3	PTZ, Electro, Other
Allopurinol	-0.420355463	Gout; renal stones	1966	1	26774005	1	KA
Methotrexate	-0.409519203	Rheumatoid arthritis; crohn's disease; psoriasis	1953	1	1448429	1	PTZ
Thalidomide	-0.405694736	Malignant disease; immunosuppression	1998	3	17449064, 21592729, 24735834	2	PTZ, Kin
Triamterene	-0.40050826	Oedema	1964	1	26855365	2	PTZ, Electro
Tacrolimus	-0.392643295	Organ rejection; atopic eczema	1994	1	25580467	1	Other
Pheniramine	-0.387844428	Hay fever; urticaria; allergic conjunctivitis	1994	1	1874553	1	Electro

Supplementary Table 4: Examples of significant drug repurposing candidates identified though connectivity mapping analysis of all epilepsy GWAS-imputed transcriptome. 30 drugs predicted to significantly reverse epilepsy-associated gene expression changes. These drugs have published evidence of antiepileptic efficacy in animal models and are already licensed for the treatment of other human diseases. The drugs are listed in order of decreasing (less negative) cosine distance values. A higher (more negative) cosine distance indicates greater likelihood of antiepileptic efficacy. Animal model evidence and drug indications and use data were extracted from the Prescribable Drugs with Efficacy in Experimental Epilepsies (PDE3) database. AUD: audiogenic; electro: electroshock; KA: kainic acid; kin: kindling; pilo: pilocarpine; PTZ: pentylenetetrazol.

	**Source of summary statistics**	**Sample size**	**All epilepsy**	**Focal epilepsy**	**Genetic generalized epilepsy**
**Headache**	Meng et al., 2018 [1]	n=223773	Rg=0.432; p=0.033	Rg=0.213; p=0.0015	Rg=0.083; p=0.049
**Alzheimer's Disease**	Lambert et al., 2013 [2]	n=74046	Rg=0.16; p=0.21	Rg=0.289; p=0.33	Rg=0.042; p=0.615
**Parkinson's Disease**	Simón-Sánchez et al., 2009 [3]	n=5691	Rg=0.089; p=0.347	Rg=0.163; p=0.351	Rg=0.004; p=0.934

**Ischaemic stroke**	ISGC, 2016 [4]	n=49323	Rg=0.4678; p=0.092	Rg=0.3172; p=0.014	Rg=0.1027; p=0.306
**Intracranial aneurysm**	van't Hof et al., 2016 [5]	n=5821	Rg=0.735; p=0.223	Rg=0.279; p=0.234	Rg=-0.059; p=0.693
**Febrile Seizures***	Feenstra et al., 2014 [6]	n=6117	Rg=0.085; p=0.785	Rg=0.169; p=0.29	Rg=0.144; p=0.195
**Migraine with aura****	Gormley et al., 2016 [7]	n=6332	Rg=0.259; p=0.274	Rg=0.172; p=0.17	Rg=0.171; p=0.07
**Migraine without aura****	Gormley et al., 2016 [7]	n=8348	Rg=0.219; p=0.333	Rg=0.07; p=0.493	Rg=-0.025; p=0.723
**Schizophrenia**	Ripke et al., 2014 [8]	n=150064	Rg=0.0597; p=0.263	Rg=0.111; p=0.459	Rg=0.009; p=0.842
**Autism spectrum disorder**	Grove et al., 2017 (Biorxiv)	n=46350	Rg=-0.294; p=0.093	Rg=-0.149; p=0.096	Rg=-0.059; p=0.349
**Bipolar disorder**	Sklar et al., 2011 [9]	n=16731	Rg=-0.047; p=0.521	Rg=-0.066; p=0.633	Rg=-0.023; p=0.724
**Major depressive disorder**	Ripke et al., 2013 [10]	n=18759	Rg=-0.039; p=0.721	Rg=-0.039; p=0.858	Rg=-0.152; p=0.086
**ADHD**	Middeldorp et al., 2016 [11]	n=17666	Rg=0.31; p=0.069	Rg=0.4588; p=0.176	Rg=0.214; p=0.120
**Systemic lupus erythematosus**	Bentham et al., 2015 [12]	n=23210	Rg=0.331; p=0.003	Rg=0.459; p=0.059	Rg=0.234; p=0.003
**Multiple sclerosis**	Sawcer et al., 2011 [13]	n=27148	Rg=0.235; p=-0.227	Rg=0.335; p=0.354	Rg=0.104; p=0.399
**Cognitive ability**	Sniekers et al., 2017 [14]	n=78308	Rg=-0.331; p=1.58E-6	Rg=-0.534; p=0.004	Rg=-0.181; p=3.0E-4
**Neuroticism**	van den Berg et al., 2014 [15]	n=160958	Rg=0.130; p=0.381	Rg=-0.052; p=0.875	Rg=0.063; p=0.22
**Openness to experience**	de Moor et al., 2012 [16]	n=17375	Rg=0.0537; p=0.684	Rg=0.252; p=0.370	Rg=-0.0836; p=0.432
**Conscientiousness**	de Moor et al., 2012 [16]	n=17375	Rg=0.0728; p=0.6844	Rg=0.193; p=0.591	Rg=0.0011; p=0.994

Supplementary Table 5: The genetic correlations of epilepsy with other phenotypes*, calculated using LD-score regression. Rg: genetic correlation coefficient. *Combined MMR-related and MMR-unrelated febrile seizures. **Migraine results without 23andMe data*

**Contributor**	**Ancestry**	**Description**	**n**	**Platform Chip**	**Institutional Review Board**
BRAINN/UNICAMP	Brazilian (Afr&Cauc)	Case	177	Affymetrix 6.0	Research Ethics Committee at the University of Campinas (UNICAMP), Brazil
		Control	163		
Philadelphia Cohort	US (Afr&Cauc)	Case	1,734	Illumina 550, Omni-Express	Thomas Jefferson University Hospital Philadelphia PA USA;<br>The Children's Hospital of Philadelphia, Philadelphia PA USA;<br>The University of Pensylvania, Philadelphia PA USA;<br>The University of Cincinnati, Cincinnati Ohio USA;<br>Nationwide Children's Hospital, Columbus Ohio, USA;<br>Beth Israel Deaconess Medical Center, Boston MA;<br>The University of Montreal, Montreal Quebec, CA
		Control	9,337		
Duke University	US (Cauc)	Case	777	Illumina 610	Duke University Institutional Review Board, Durham, NC, USA
EPGP	US (Cauc)	Case	1,298	Illumina HumanCore	Montefiore Medical Center Institutional Review Board;<br>Hospital General de Agudos J.M.Ramos Mejia Comite de Etica en Investigacion;<br>Children's Hospital Boston Office of Clinical Investigation;<br>University of Pittsburgh Institutional Review Board;<br>Children's Hospital of Philadelphia Commitees for Protection of Human Subjects;<br>Cincinnati Children's Hospital Medical Center Institutional Review Board;<br>Cleveland Clinic Institutional Review Board;<br>Columbia University Medical Center;<br>Colorado Multiple Institutional IRB Board;<br>Emory University Institutional Review Board;

					University of Texas Health Science Center at Houston Committee for the Protection of Human Subjects;<br>Jefferson University Institutional Review Board;<br>Johns Hopkins Medicine Institutional Review Boards;<br>Mayo Clinic Institutional Review Boards;<br>Austin Health Human Research Ethics Committee;<br>University of Michigan Institutional Review Board;<br>McGill University Health Centre Research Ethics Board;<br>Louisiana Health Sciences Center New Orleans Institutional Review Board;<br>Upper South B Regional Ethics Committee, Ministry of Health;<br>NYU School of Medicine Institutional Review Board;<br>Rush University Medical Center Institutional Review Board;<br>Seattle Children's Hospital Institutional Review Board;<br>Saint Barnabas Medical Center Institutional Review Board;<br>University of Alabama at Birmingham Institutional Review Board;<br>University of California, San Francisco Committee on Human Research;<br>Vanderbilt University Institutional Review Board;<br>University of Virginia Institutional Review Board for Health Sciences Research;<br>Washington University in St. Louis Human Research Protection Office
EPICURE SP1	NW European	Case	1,495	Affymetrix 6.0	Ethics Committee at the Medical Faculty of the Eberhard-Karls University and the University Hospital Tübingen;<br>University Hospital of Bonn Ethical Committee;<br>Medical Ethical Board of the University Medical Center Utrecht, The Netherlands
EPICURE SP5	NW European	Case	788	Illumina Omni-Express	Ethics Committee at the Medical Faculty of the Eberhard-Karls University and the University Hospital Tübingen;<br>University Hospital of Bonn Ethical Committee;<br>Medical Ethical Board of the University Medical Center Utrecht, The Netherlands
EpiPGX	NW European	Case	5,031	Illumina Omni-Express	Joint Research Ethics Committee, National Hospital for Neurology and Neurosurgery and Institute of Neurology, 00/N081 NRES Committee London - Camden & Islington;<br>North West 3 Research Ethics Committee in April 2010 (ref: 10/H1002/5);<br>North-West Multicentre Research Ethics Committee (ref: MREC 02/8/45);<br>West Ethics Committee, North Glasgow University Hospitals NHS Trust (ref: 02/119(2); METC 09-352K: The Genetics of epilepsy;<br>Beaumont Hospital Ethics Committee (study code 02/44;<br>University Hospital of Bonn Ethical Committee
GenEpa	Finnish	Case	422	Illumina 610	Suomen valtakunnallinen lääketieteellinen tutkimuseettinen toimikunta TUKIJA
		Control	293		
German controls	German	Control	1,317	Illumina OmniExpress	University Hospital of Bonn Ethical Committee (040/07)
Helsinki Birth Cohort	Finnish	Control	1,586	Illumina 610	Coordinating Ethics Committee of The Hospital District of Helsinki and Uusimaa
KORA	German	Control	1,331	Affymetrix 6.0	Ethics Committee of the Bavarian Medical Association (Bayerische Landesärztekammer)
Imperial-Liverpool-Melbourne	British	Case	1,294	Illumina Omni-Express	North West Multi-centre Research Ethics Committee, Manchester, UK
Northwest-Europeans	Belgian	Control	1,622	Illumina HH300	The Erasmus-ULB Ethics Committee, Brussels
POBI	British	Control	2,706	Illumina 1.2M	NRES Committee, Yorkshire and the Humber – Leeds West, UK (Reference 05/Q1205/35)
PopGen	NW European	Control	1,062	Affymetrix 6.0	The ethics commission of the Faculty of Medicine of the Christian-Albrechts University of Kiel (CAU)
RCSI	Irish	Case	645	Illumina 610	Beaumont Hospital Ethics Committee;<br>St James Hospital and Adelaide and Meath Hospital Research Ethics Committee

Royal Melbourne Hospital	Australian (Cauc)	Case	348	Illumina 610, Omni-Express	Melbourne Health Human Research and Ethics Committee
Trinity Student Study	Irish	Control	2,232	Illumina Omni1-Quad	Dublin Federated Hospitals Research Ethics Committee
UCL	British	Case	1,051	Illumina 610	NRES Committee London – Camden & Islington
UK National Blood Service	British	Control	2,501	Illumina 1.2M	NRES Committee London – Camden & Islington
ULB	Belgian	Case	539	Illumina 610	The Erasmus-ULB Ethics Committee, Brussels
University of Bonn	German	Case	284	Affymetrix 6.0	University Hospital of Bonn Ethical Committee (040/07)
University of Hong Kong	Han Chinese	Case	533	Illumina 550, 610	The Joint Chinese University of Hong Kong – New Territories East Cluster Clinical Research Ethics Committee
		Control	2,875		
Wellcome 1958 Birth Cohort	British	Control	2,699	Illumina 1.2M	North West – Haydock Research Ethics Committee; NRES Committee London – Camden & Islington

Supplementary Table 6: Details of participating cohorts. *Numbers of cases and controls are after quality control filtering.*

Phenotype	Ethnicity	λ	λ_{1000}	Mean χ2	LDSC intercept
**All epilepsy**	Caucasian	1.2514	1.0138	1.175	1.151
	African-American	0.9977	0.9917	0.995	1.0122
	Asian	1.045	1.05	1.047	1.0138
**Focal epilepsy**	Caucasian	1.197	1.0176	1.283	1.18
	African-American	0.9977	0.9862	0.994	0.9987
	Asian	1.045	1.0537	1.04	1.0231
**Generalized epilepsy**	Caucasian	1.2514	1.04255	1.296	1.098
	African-American	0.9977	0.9807	0.994	1.0174
**CAE**	Caucasian	1.094	1.0636	1.136	1.068
**JAE**	Caucasian	1.094	1.11648	1.085	1.037
**JME**	Caucasian	1.1447	1.06644	1.156	1.05
**GTCS alone**	Caucasian	1.045	1.1015	1.063	1.025
**Focal-HS**	Caucasian	1.094	1.0695	1.115	1.078
**Focal, other lesion**	Caucasian	1.094	1.0203	1.092	1.094
**Focal lesion negative**	Caucasian	1.094	1.0209	1.091	1.077

Supplementary Table 7: Estimation of inflation factor and the LD-score regression intercept *stratified by ethnicity and phenotype. λGC: genomic inflation factor; λ1000: genomic inflation factor corrected for an equivalent study of 1000 cases and 1000 controls.*

ADSL	*CHRNA2*	*CSTB*	*GABRA1*	*GRIN2B*	*KCNQ2*	*NHLRC1*	*PPT1*	*SCN9A*	*STX1B*	*WWOX*
ALDH7A1	*CHRNA4*	*CTSD*	*GABRB2*	*HCN1*	*KCNQ3*	*NR2F1*	*PRICKLE1*	*SIK1*	*STXBP1*	*ZEB2*
ALG13	*CHRNA7*	*DEPDC5*	*GABRB3*	*HNRNPU*	*KCNT1*	*NRXN1*	*PRICKLE2*	*SLC13A5*	*SYNGAP1*	
ARHGEF9	*CHRNB2*	*DNAJC5*	*GABRG2*	*IQSEC2*	*KCTD7*	*PCDH19*	*PRRT2*	*SLC25A22*	*TBC1D24*	
ARX	*CLN2*	*DNM1*	*GAMT*	*KANSL1*	*LGI1*	*PIGA*	*QARS*	*SLC2A1*	*TCF4*	
ATP1A2	*CLN3*	*DYRK1A*	*GATM*	*KCNA2*	*MAGI2*	*PIGO*	*SCARB2*	*SLC35A2*	*TPP1*	
ATP6AP2	*CLN5*	*EEF1A2*	*GNAO1*	*KCNB1*	*MBD5*	*PIGV*	*SCN1A*	*SLC6A1*	*TSC1*	
CACNA1A	*CLN6*	*EPM2A*	*GOSR2*	*KCNC1*	*MECP2*	*PNKP*	*SCN1B*	*SLC6A8*	*TSC2*	
CDKL5	*CLN8*	*FOLR1*	*GRIN1*	*KCNJ10*	*MEF2C*	*PNPO*	*SCN2A*	*SLC9A6*	*UBE3A*	
CHD2	*CNTNAP2*	*FOXG1*	*GRIN2A*	*KCNMA1*	*MFSD8*	*POLG*	*SCN8A*	*SPTAN1*	*WDR45*	

Supplementary Table 8: List of selected monogenic epilepsy genes *that were tested for enrichment in our GWAS. We supplemented the list of 43 known dominant epilepsy genes (Epi4K Consortium, 2017) with an additional 59 monogenic epilepsy genes from the GeneDX comprehensive epilepsy panel (www.genedx.com).*

	Number of Datasets	Dataset names					
BCL11A	6	Hawrylycz	Miller	Kang	Hernandez	Trabzuni	Zhang
GJA1	6	Hawrylycz	Miller	Kang	Colantuoni	Hernandez	Zhang
GLS	5	Hawrylycz	Miller	Colantuoni	Hernandez	Trabzuni	
GTDC1	5	Hawrylycz	Miller	Kang	Hernandez	Zhang	
CSRNP3	4	Hawrylycz	Miller	Kang	Trabzuni		
PRSS36	4	Hawrylycz	Kang	Colantuoni	Trabzuni		
PTPRK	4	Hawrylycz	Kang	Hernandez	Zhang		
VKORC1	4	Miller	Hernandez	Trabzuni	Zhang		
ATXN1	3	Hawrylycz	Colantuoni	Hernandez			
CTF1	3	Kang	Colantuoni	Trabzuni			
FBRS	3	Hawrylycz	Miller	Colantuoni			
GABRA2	3	Hawrylycz	Miller	Colantuoni			
GABRG1	3	Hawrylycz	Miller	Colantuoni			
KCNAB1	3	Miller	Colantuoni	Zhang			
LRRC46	3	Hawrylycz	Kang	Colantuoni			
PRSS8	3	Hawrylycz	Miller	Kang			
PYDC1	3	Hawrylycz	Hernandez	Zhang			
ZNF646	3	Miller	Colantuoni	Trabzuni			
BCKDK	2	Miller	Zhang				
CBX1	2	Hawrylycz	Miller				
FBXL19	2	Hawrylycz	Miller				
FKBP1B	2	Miller	Zhang				
HOXB13	2	Hawrylycz	Colantuoni				
HOXB5	2	Kang	Colantuoni				
HOXB9	2	Kang	Trabzuni				
HSD3B7	2	Trabzuni	Zhang				
KRTAP19-1	2	Hawrylycz	Miller				
KRTAP8-1	2	Hawrylycz	Miller				
MRPL10	2	Miller	Colantuoni				
OSBPL7	2	Colantuoni	Trabzuni				
PCDH7	2	Colantuoni	Hernandez				
PRR14	2	Hernandez	Trabzuni				
PYCARD	2	Miller	Hernandez				
SCN3A	2	Hawrylycz	Kang				
SCRN2	2	Trabzuni	Zhang				
SP6	2	Hawrylycz	Kang				
STAT4	2	Colantuoni	Hernandez				
TBKBP1	2	Hawrylycz	Miller				
TBX21	2	Kang	Trabzuni				
TP53I3	2	Hawrylycz	Miller				
VRK2	2	Hawrylycz	Trabzuni				
ZNF629	2	Hawrylycz	Zhang				
ZNF689	2	Kang	Trabzuni				
ADCY7	1	Zhang					

BRD7	1	Hawrylycz					
CCDC112	1	Miller					
CDK5RAP3	1	Hernandez					
COPZ2	1	Zhang					
GNPDA2	1	Zhang					
GRIK1	1	Miller					
HIBCH	1	Zhang					
HOXB1	1	Kang					
HOXB2	1	Colantuoni					
HOXB3	1	Kang					
HOXB4	1	Kang					
HOXB7	1	Hawrylycz					
HOXB8	1	Kang					
KAT8	1	Miller					
KCNN2	1	Colantuoni					
KLHL29	1	Hernandez					
KRTAP13-3	1	Colantuoni					
KRTAP15-1	1	Colantuoni					
KRTAP19-6	1	Colantuoni					
KRTAP19-7	1	Colantuoni					
KRTAP21-1	1	Zhang					
KRTAP21-2	1	Kang					
KRTAP6-3	1	Hawrylycz					
MFSD2B	1	Hawrylycz					
MFSD6	1	Hernandez					
NAB1	1	Hernandez					
NFE2L1	1	Hernandez					
NPEPPS	1	Miller					
PAPD5	1	Miller					
PLCH1	1	Zhang					
RNF40	1	Zhang					
SCN7A	1	Miller					
SETD1A	1	Hernandez					
SLC33A1	1	Kang					
SNX11	1	Kang					
STX4	1	Hernandez					
THEMIS	1	Hawrylycz					
TRIM36	1	Hernandez					
TRIM72	1	Zhang					
ZNF423	1	Kang					
ZNF668	1	Trabzuni					

Supplementary Table 9: brain-coX in silico gene co-expression prioritization *results with 146 candidate epilepsy genes identified from the GWAS. 85 genes were prioritised in at least one brain gene expression datasets. First author name of paper describing relevant datasets for each gene are given as columns.*

CA12	*SCN7A*	*SCN10A*	*CACNA1G*	*GRIA3*	*GRIK1*	*GABRQ*	*GRIN2C*
CA1	*SCN5A*	*GABRA1*	*CACNA1H*	*GRIA4*	*GABRA4*	*ALDH5A1*	*GRIN3A*
CA2	*CHRNA2*	*GABRG2*	*CACNA1I*	*GABRB1*	*GABRE*	*ABAT*	*CHRNA4*
CA4	*SCN9A*	*GABRA5*	*CACNA2D1*	*GABRB3*	*GRIK2*	*GRIN2D*	*CHRNA7*
CHRNB4	*SCN2A*	*GABRA3*	*CACNA1B*	*GABRB2*	*GRIK3*	*GRIN3B*	*KIT*
SLC6A4	*SCN3A*	*GABRA2*	*SV2A*	*SLC6A1*	*GRIK4*	*GRIN1*	*PDGFRA*
SCN1A	*SCN11A*	*GABRG1*	*GRIA1*	*GABRP*	*GABRA6*	*GRIN2A*	*PDGFRB*
SCN4A	*SCN8A*	*GABRG3*	*GRIA2*	*GABRD*	*GRIK5*	*GRIN2B*	*SLC18A2*

Supplementary Table 10: List of genes that are targets of currently used anti-epileptic drugs *which were tested for enrichment in our GWAS. We compiled the list of drug target genes from (Santos et al., 2017), supplemented with additional FDA & EMA licensed AEDs.*

**Phenotype**	**Cases**	**Controls**	**P (proportion of cases in sample)**	**K (disease prevalence)**	**Z**	**h_o^2 (LDAK observed heritability)**	**h_L^2: liability scale heritability (95% CI)**	**h_L^2: liability scale heritability modeled with half prevalence (K/2)**	**h_L^2: liability scale heritability modeled with double prevalence (K*2)**
**All epilepsy**	1,4534	24,218	0.3751	0.005	0.01446	0.191586	0.097 (0.091 - 0.11)	0.084	0.11
**Focal epilepsy**	9,095	24,218	0.2730	0.003	0.00915	0.17111	0.092 (0.084 - 0.10)	0.082	0.11
**Generalized epilepsy**	3,708	24,218	0.1328	0.002	0.00634	0.37258	0.32 (0.30 - 0.35)	0.28	0.37
**CAE**	636	24,218	0.0256	0.00035	0.00128	0.194	0.58 (0.38 - 0.78)	0.53	0.64
**JAE**	273	24,218	0.0111	0.00015	0.00058	0.121	0.74 (0.37 - 1.11)	0.67	0.81
**JME**	1,177	24,218	0.0463	0.00015	0.00058	0.30413	0.46 (0.33 - 0.60)	0.42	0.51
**GTCS alone**	225	24,218	0.0092	0.0002	0.00076	0.140328	1.07 (0.61 - 1.54)	0.98	1.18
**Focal lesion negative**	2,660	24,218	0.0990	0.0009	0.00306	0.074626	0.072 (0.017 - 0.13)	0.065	0.082
**Focal HS**	709	24,218	0.0284	0.00075	0.00258	0.059077	0.18 (0.0 - 0.36)	0.16	0.20
**Focal, other lesion**	2,751	24,218	0.1020	0.00135	0.00443	0.04024	0.040 (0.0 - 0.091	0.036	0.046

Supplementary Table 11: SNP-based heritabilities *computed with LDAK and converted to liability scale.*

Phenotype	LDAK		BOLT-REML		LDSC	
	$\mathbf{h_o^2}$	$\mathbf{h_L^2}$ **(L95-U95)**	$\mathbf{h_o^2}$	$\mathbf{h_L^2}$ **(L95-U95)**	$\mathbf{h_o^2}$	$\mathbf{h_L^2}$ **(L95-U95)**
**All epilepsy**	0.192	0.097 (0.091-0.102)	0.227	0.115 (0.107-0.122)	0.1245	0.063 (0.043-0.082)
**Focal epilepsy**	0.171	0.092 (0.083-0.101)	0.172	0.093 (0.083-0.102)	0.0324	0.017 (-0.004-0.039)
**Genetic generalized epilepsy**	0.373	0.321 (0.296-0.345)	0.315	0.271 (0.253-0.288)	0.3217	0.277 (0.225-0.329)
**CAE**	0.194	0.584 (0.385-0.784)	0.126	0.379 (0.321-0.438)	0.1019	0.307 (0.164-0.449)
**JAE**	0.121	0.736 (0.365-1.107)	0.055	0.332 (0.226-0.439)	0.0584	0.355 (0.125-0.585)
**JME**	0.304	0.462 (0.326-0.596)	0.173	0.263 (0.232-0.294)	0.1885	0.286 (0.199-0.373)
**GTCS alone**	0.14	1.071 (0.607-1.535)	0.073	0.558 (0.416-0.700)	0.0735	0.561 (0.248-0.874)
**Focal lesion negative**	0.075	0.072 (0.017-0.128)	0.091	0.089 (0.072-0.105)	0.0239	0.023 (-0.015-0.061)
**Focal HS**	0.059	0.18 (-0.004-0.363)	0.161	0.491 (0.431-0.551)	0.0414	0.126 (0.036-0.216)
**Focal, other lesion**	0.04	0.041 (-0.009-0.091)	0.088	0.089 (0.072-0.106)	-0.0021	-0.002 (-0.043-0.039)

Supplementary Table 12: Comparative SNP-based heritability estimates *computed by LDAK, BOLT-REML and LDSC.*
h_o^2: *Observed SNP-based heritability.* h_L^2: *SNP-based heritability converted to liability scale.*

Supplementary Figures

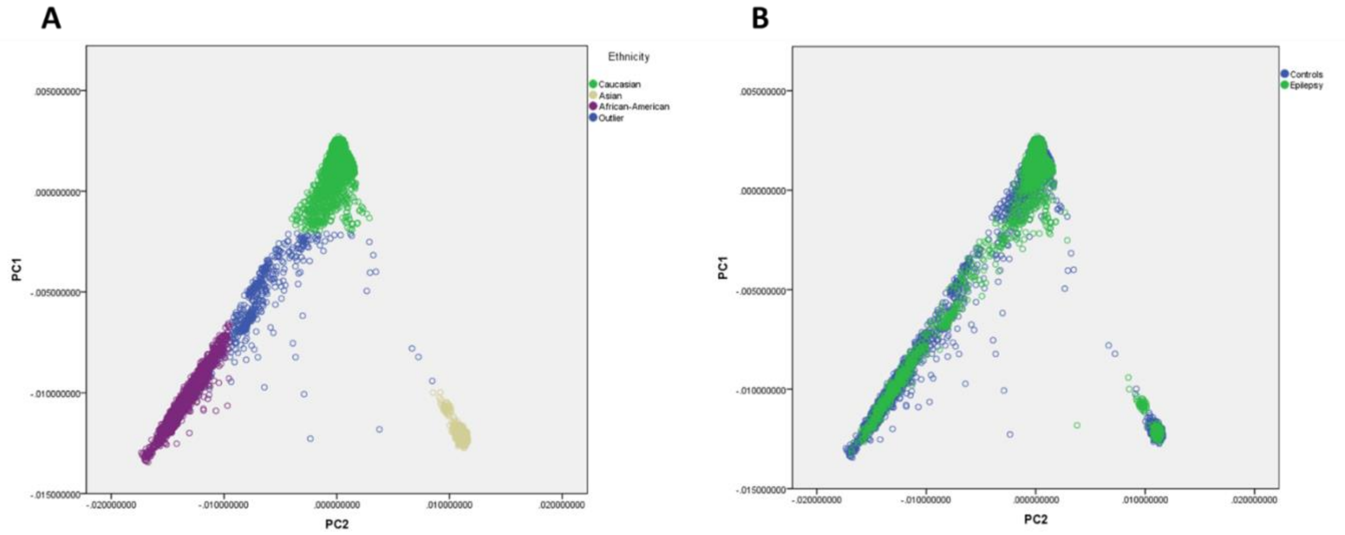

Supplementary Figure 1: Principal component plots. *(**A**) Three different ethnicities and outliers inferred by principal components. (**B**) Principal component plot with colours stratified by epilepsy cases and controls.*

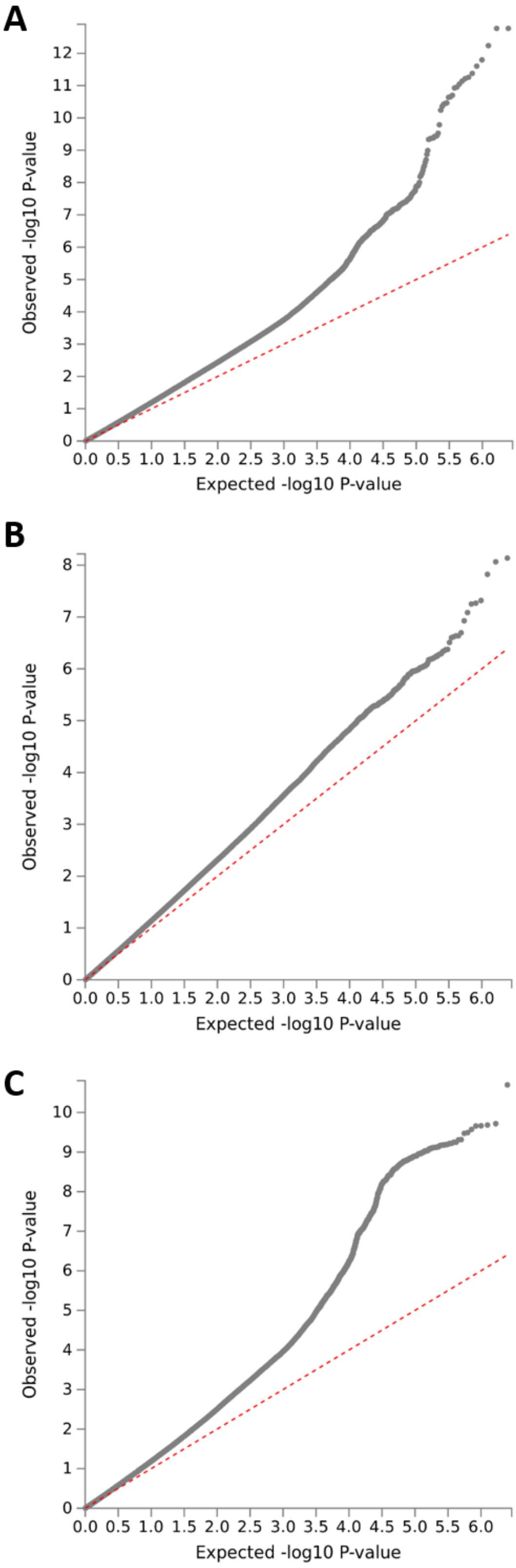

Supplementary Figure 2: Quantile-quantile (Q-Q) plots. Q-Q plots of meta-analyses *of (A) all epilepsy (λ=1.25), (B) focal epilepsy (λ=1.18) and (C) genetic generalized epilepsy (λ=1.24).*

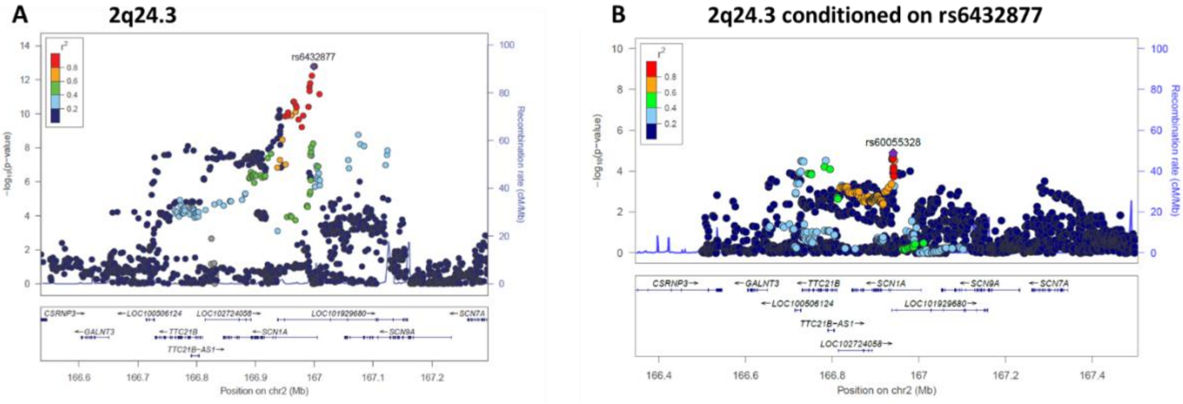

Supplementary Figure 3: Conditional analysis of 2q24.3 *shows two independent signals associated with all epilepsy. The same 2q24.3 locus is shown before (**A**) and after (**B**) conditioning on the lead SNP in the locus (rs6432877). Negative log10-transformed P-values (Y-axis) are plotted against chromosomal position (x-axis). The conditional threshold for significance was set at 2x10^{-5}, based on approximately 2,500 imputed variants per 1MB region.*

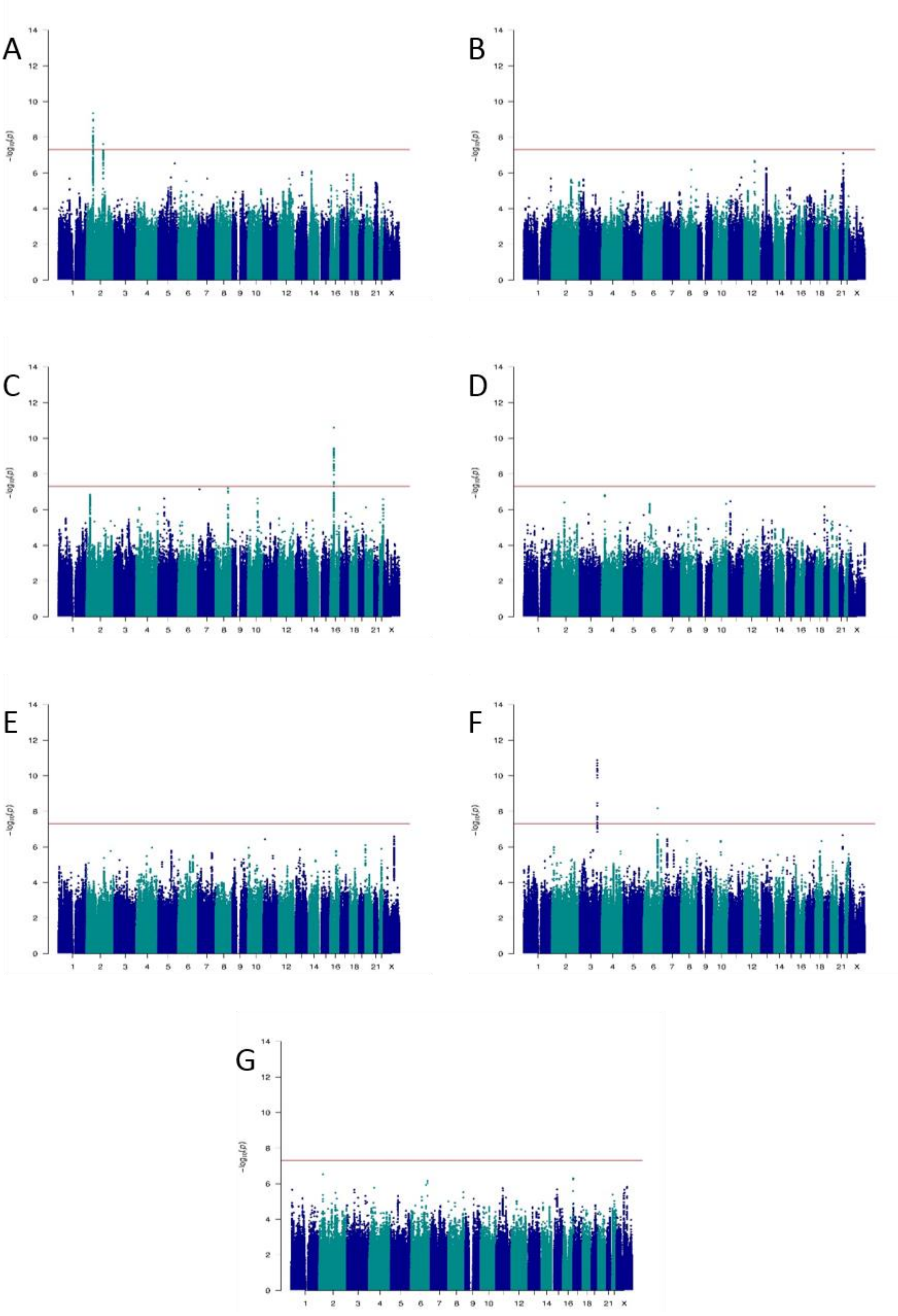

Supplementary Figure 4: Manhattan plots for the 7 epilepsy subtypes. *Chromosomal position is plotted on the X-axis and -log10 transformed P-values are plotted on the Y-axis. A. childhood absence epilepsy; B. juvenile absence epilepsy; C. juvenile myoclonic epilepsy; D. generalized tonic-clonic seizures alone; E. focal epilepsy, lesion negative; F. focal epilepsy with hippocampal sclerosis; G. focal epilepsy, other lesion*

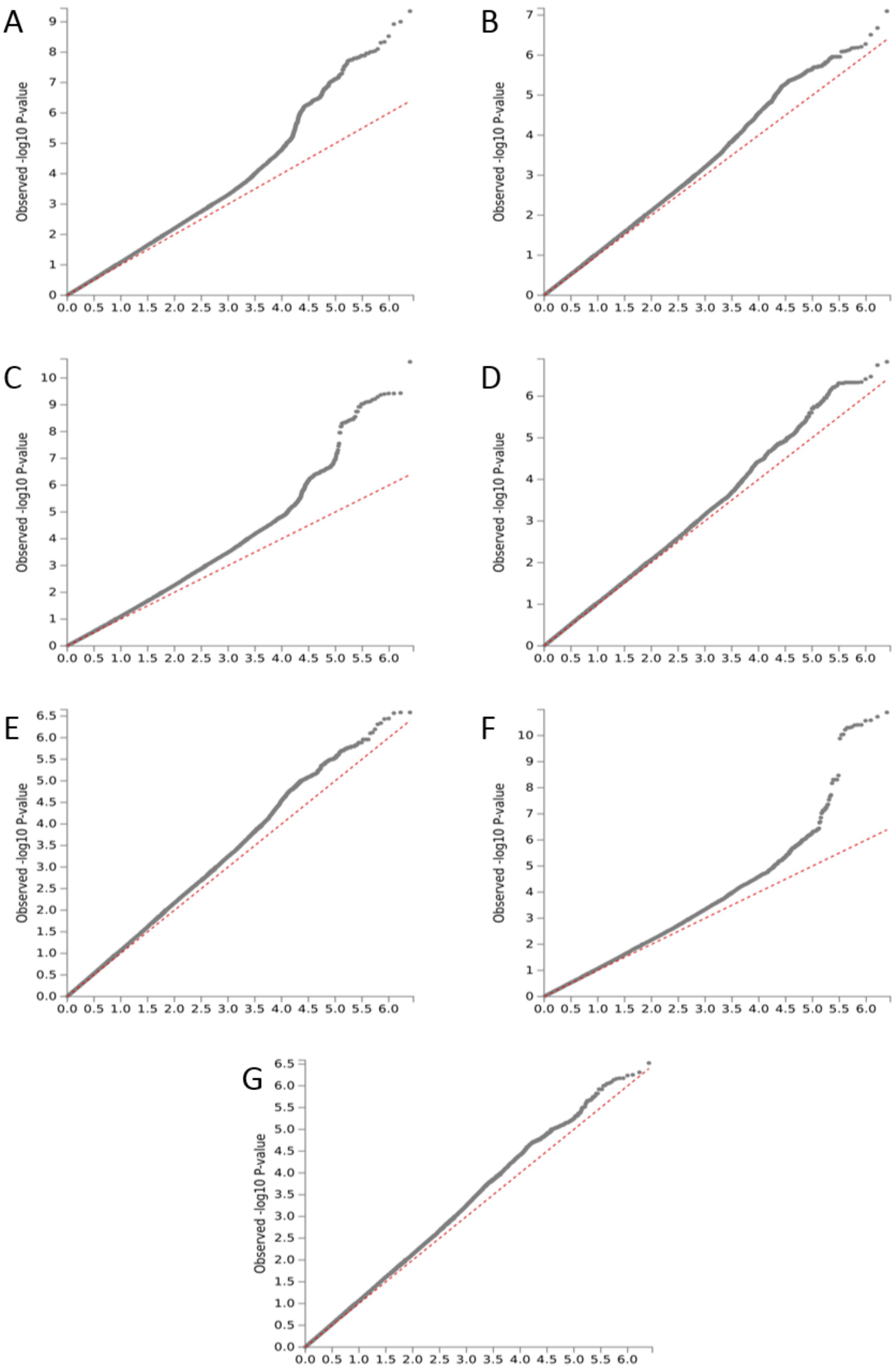

Supplementary Figure 5: Q-Q plots of the 7 epilepsy subtypes. *A. childhood absence epilepsy; B. juvenile absence epilepsy; C. juvenile myoclonic epilepsy; D. generalized tonic-clonic seizures alone; E. focal epilepsy, lesion negative; F. focal epilepsy with hippocampal sclerosis; G. focal epilepsy, other lesion*

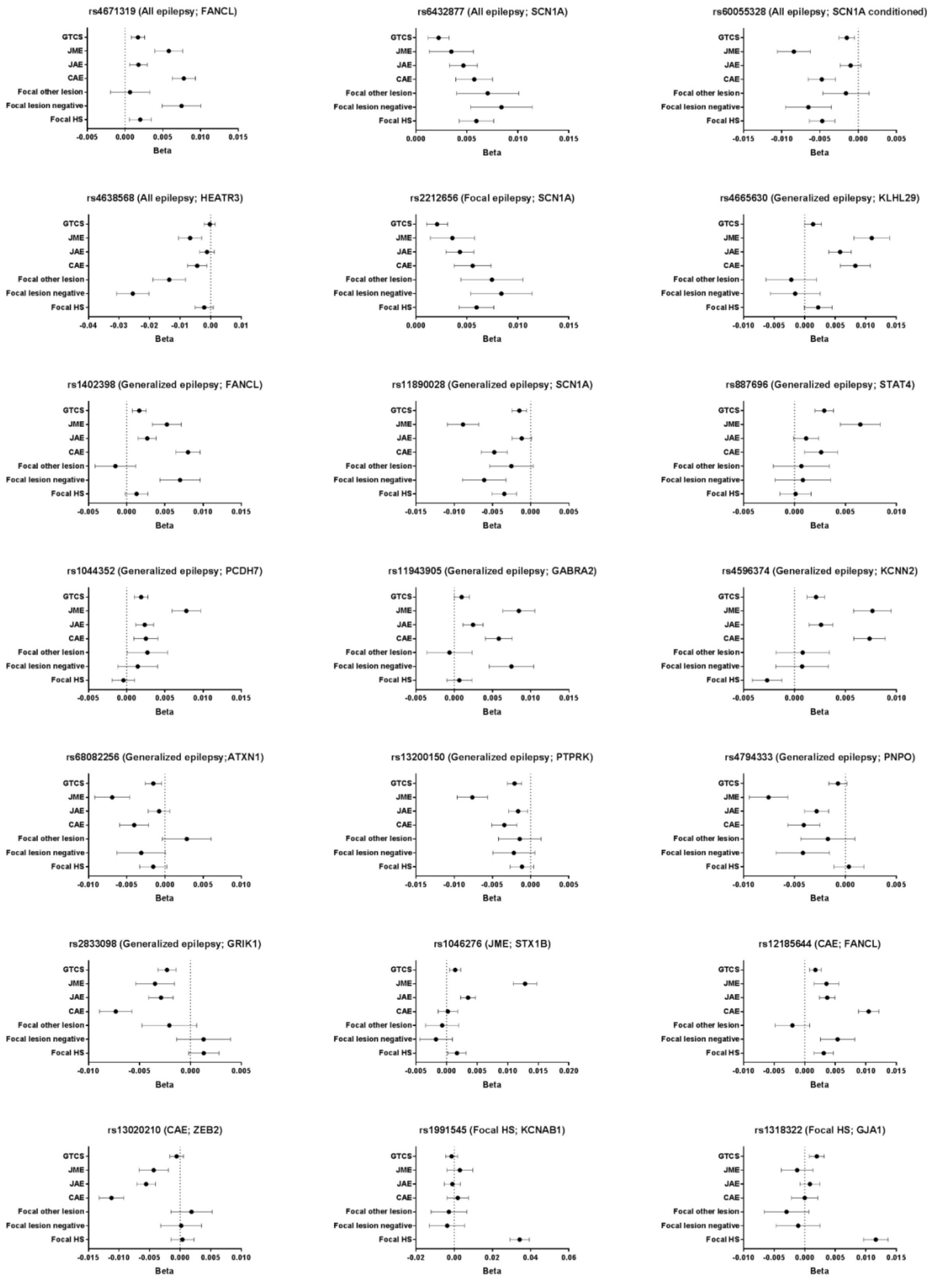

Supplementary Figure 6: Forest plot of the lead SNPs *from genome-wide significant loci in the 7 epilepsy subtypes. The subphenotype where the lead SNP was identified is displayed in parentheses, followed by a representative gene in the locus. Beta regression coefficients with standard errors from BOLT-LMM are displayed on the X-axis.*

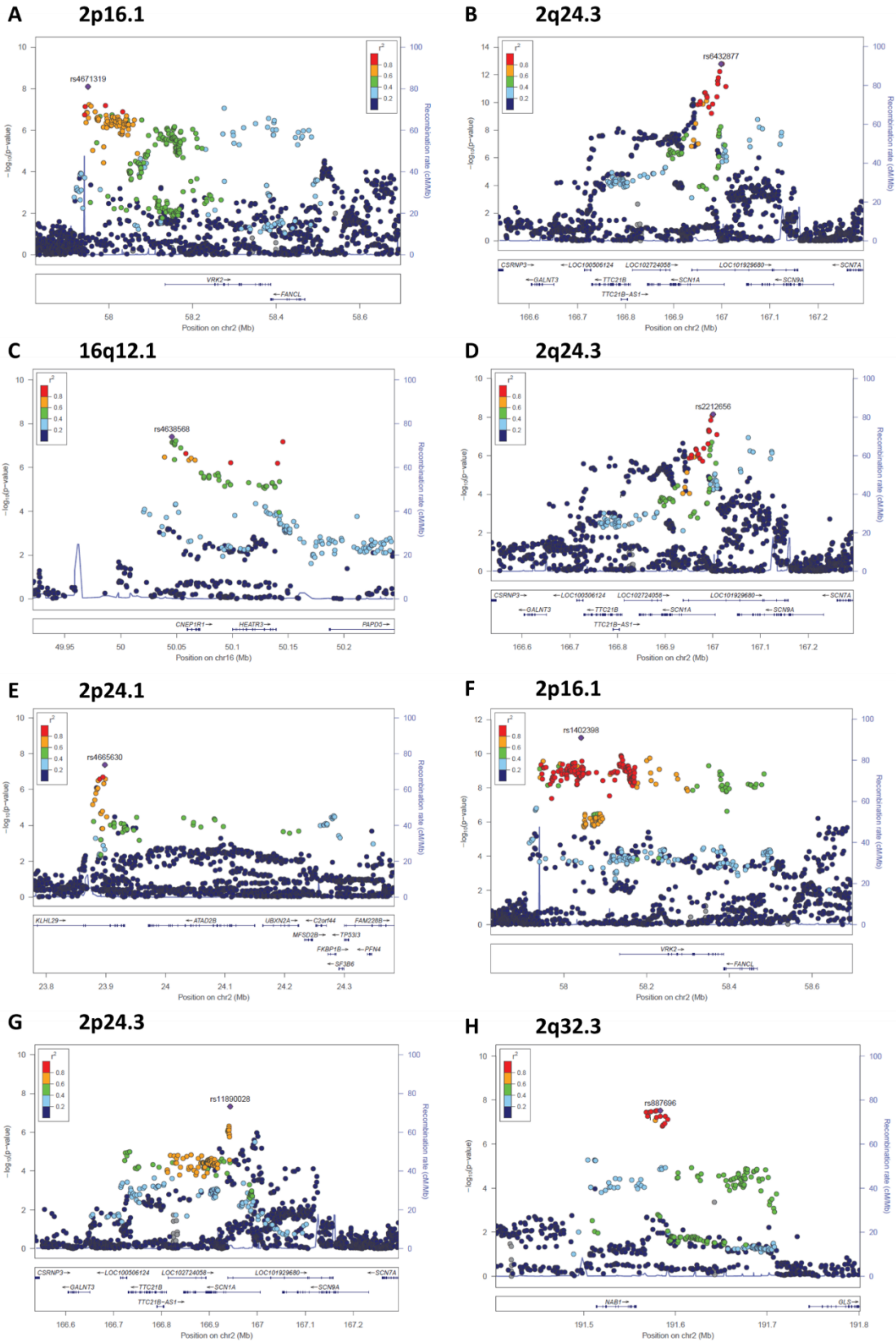

A 2p16.1
rs4671319
Position on chr2 (Mb)
B 2q24.3
rs6432877
Position on chr2 (Mb)
C 16q12.1
rs4638568
Position on chr16 (Mb)
D 2q24.3
rs2212656
Position on chr2 (Mb)
E 2p24.1
rs4665630
Position on chr2 (Mb)
F 2p16.1
rs1402398
Position on chr2 (Mb)
G 2p24.3
rs11890028
Position on chr2 (Mb)
H 2q32.3
rs887696
Position on chr2 (Mb)

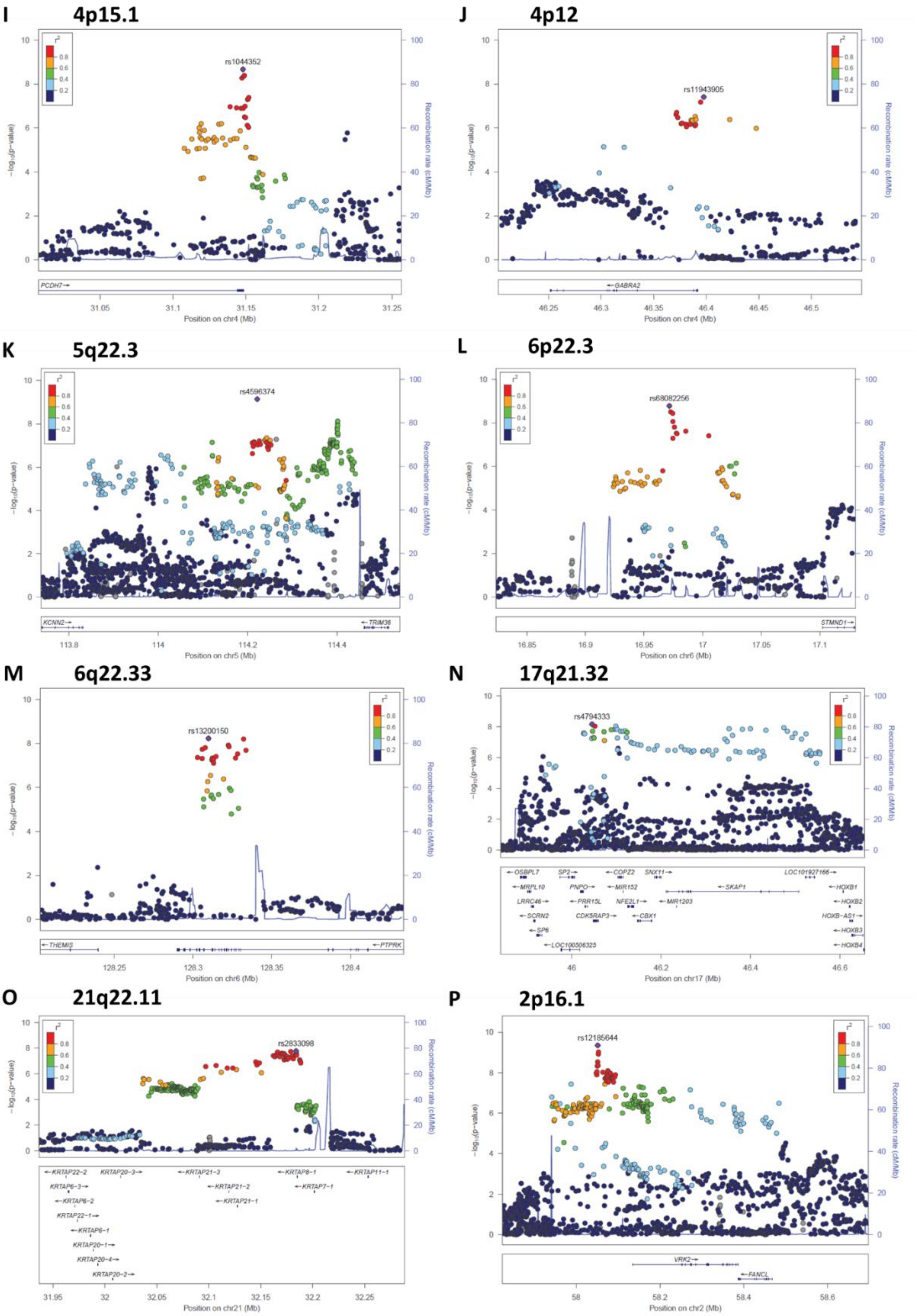
I
4p15.1
rs1044352
PCDH7
Position on chr4 (Mb)
J
4p12
rs11943905
GABRA2
Position on chr4 (Mb)
K
5q22.3
rs4596374
KCNN2
TRIM36
Position on chr5 (Mb)
L
6p22.3
rs68082256
STMND1
Position on chr6 (Mb)
M
6q22.33
rs13200150
THEMIS
PTPRK
Position on chr6 (Mb)
N
17q21.32
rs4794333
Position on chr17 (Mb)
O
21q22.11
rs2833098
Position on chr21 (Mb)
P
2p16.1
rs12185644
VRK2
FANCL
Position on chr2 (Mb)
−log10(p−value)
Recombination rate (cM/Mb)

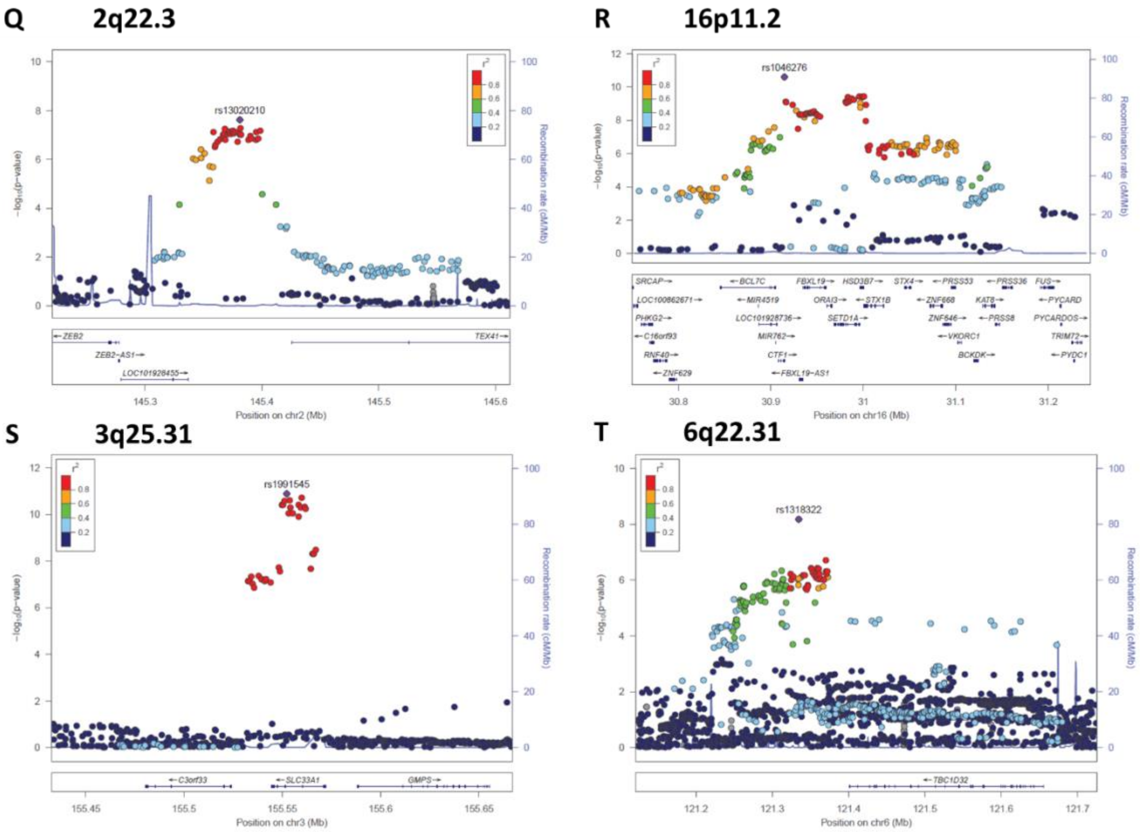

Supplementary Figure 7: Locuszoom plots for all genome-wide significant loci. Genome-wide significant loci *from (**A-C**) all epilepsy, (**D**) focal epilepsy, (**E-O**) genetic generalized epilepsy, (**P-Q**) childhood absence epilepsy, (**R**) juvenile myoclonic epilepsy, and (**S-T**) focal epilepsy with hippocampal sclerosis. SNPs are colored according to pairwise LD with respect to the lead SNP of the locus. Negative log10-transformed P-values (Y-axis) are plotted against chromosomal position (x-axis).*

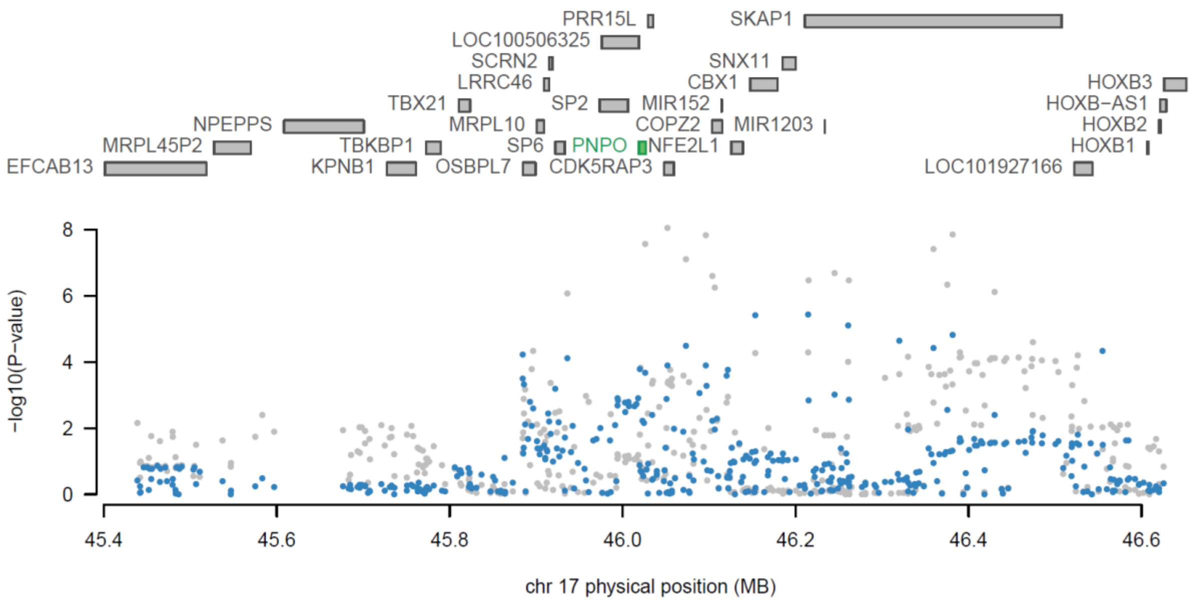

Supplementary Figure 8: GWAS results of genetic generalized epilepsy conditioned on expression of PNPO. *Expression of PNPO was imputed using TWAS based on expression QTL weights from dorsolateral prefrontal tissue (CommonMind database). Unconditional GWAS results are displayed in grey and the same SNPs conditioned on expression of PNPO are displayed in blue.*

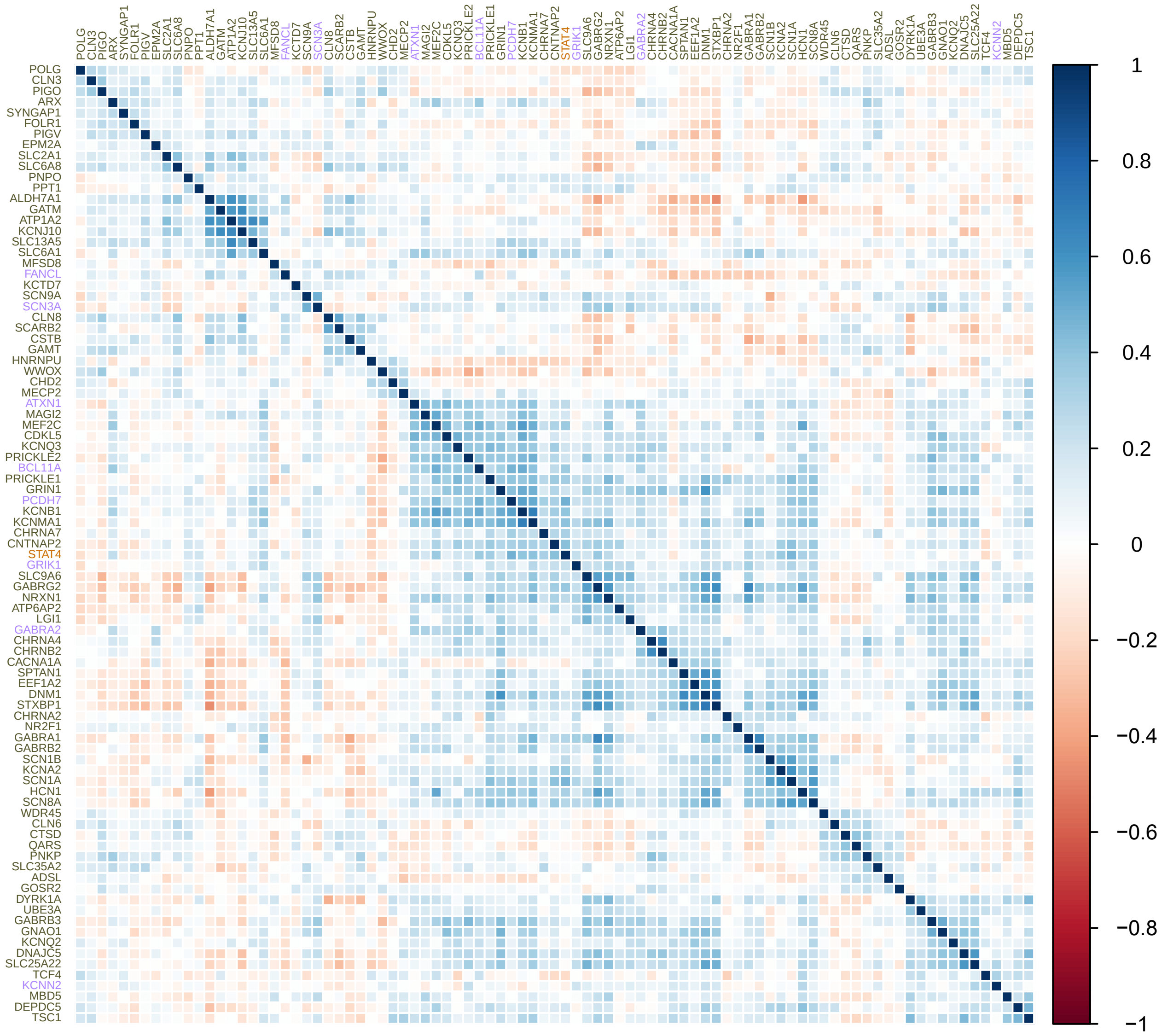

Supplementary Figure 9: Gene co-expression matrix produced by brain-coX. Average correlation over 7 brain gene expression datasets between the expression of 80 known epilepsy genes (Supplementary Table 8, displayed with black font for gene labels), 8 GWAS candidate genes (purple font) and STAT4 (red font) across 7 datasets. Known epilepsy and candidate genes are only shown if found in all datasets.

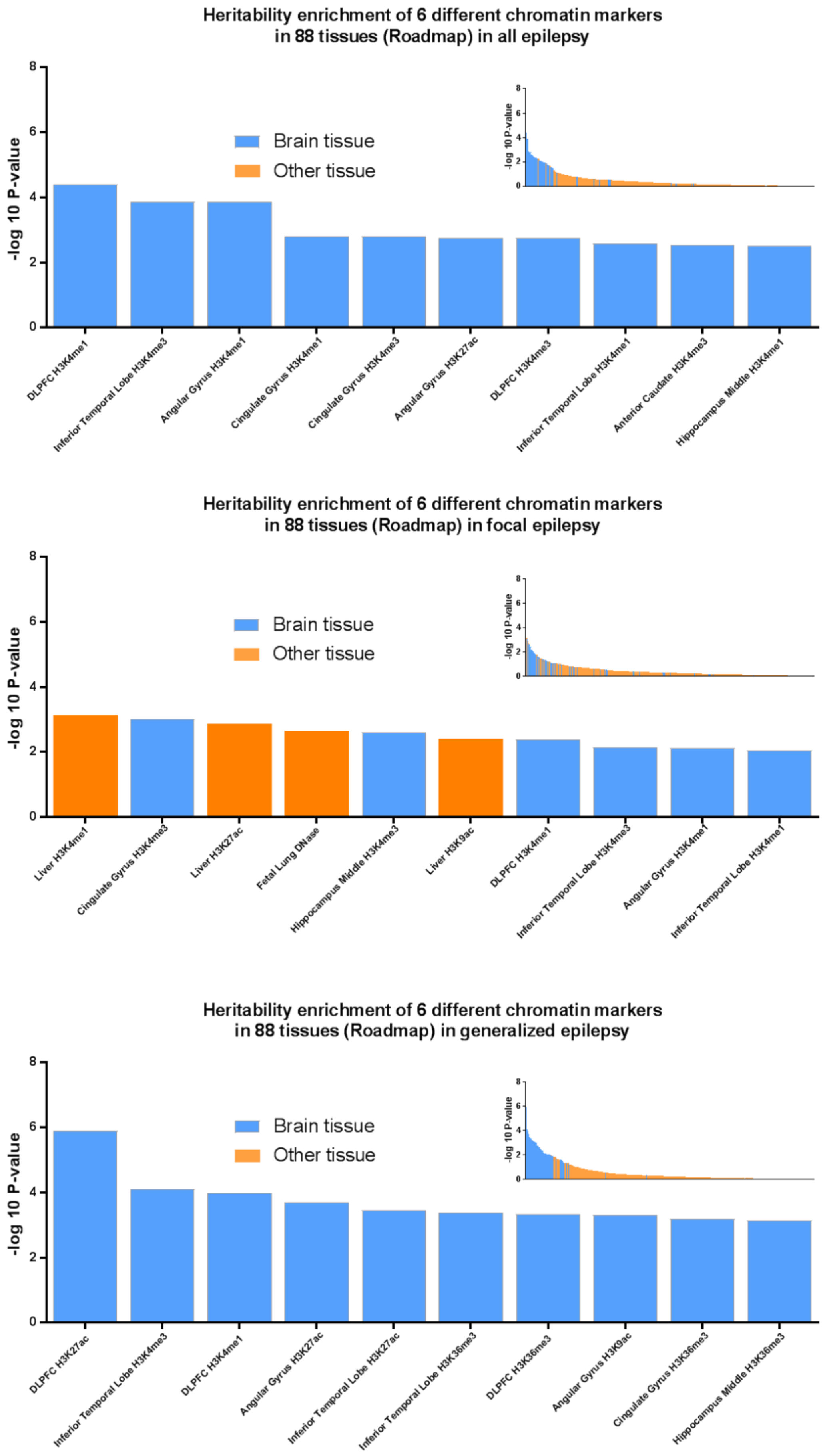

Supplementary Figure 10: Heritability enrichment of 6 different chromatin markers in 88 tissues *(data from the Roadmap Epigenomics Project) in (top) all epilepsy, (middle) focal epilepsy and (bottom) genetic generalized epilepsy GWAS.*

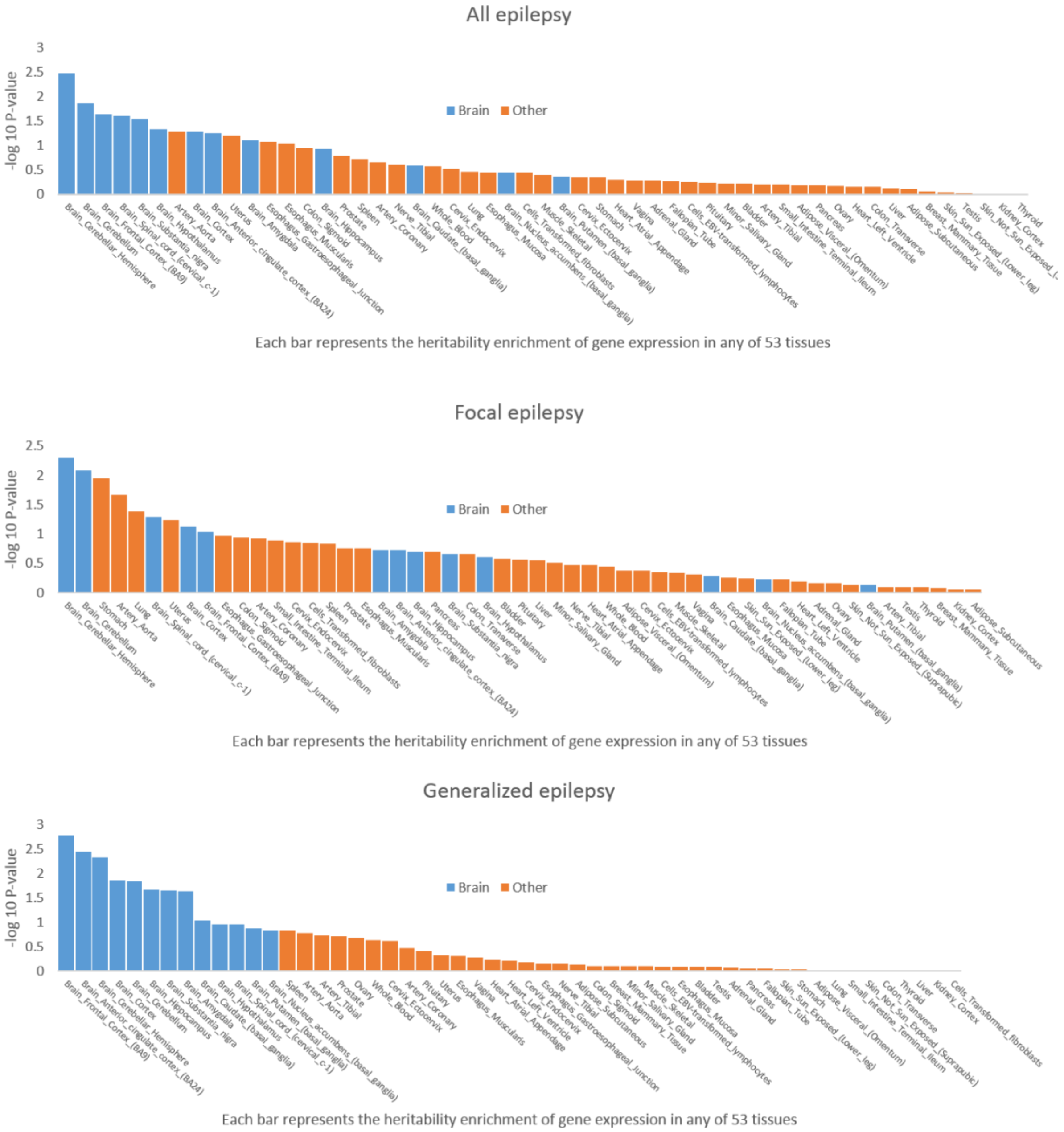

Supplementary Figure 11: Heritability enrichment of genes expressed in 53 tissues *in (top) all epilepsy, (middle) focal epilepsy and (bottom) genetic generalized epilepsy GWAS. Gene expression data from the gene-tissue expression (GTEx) consortium was used.*

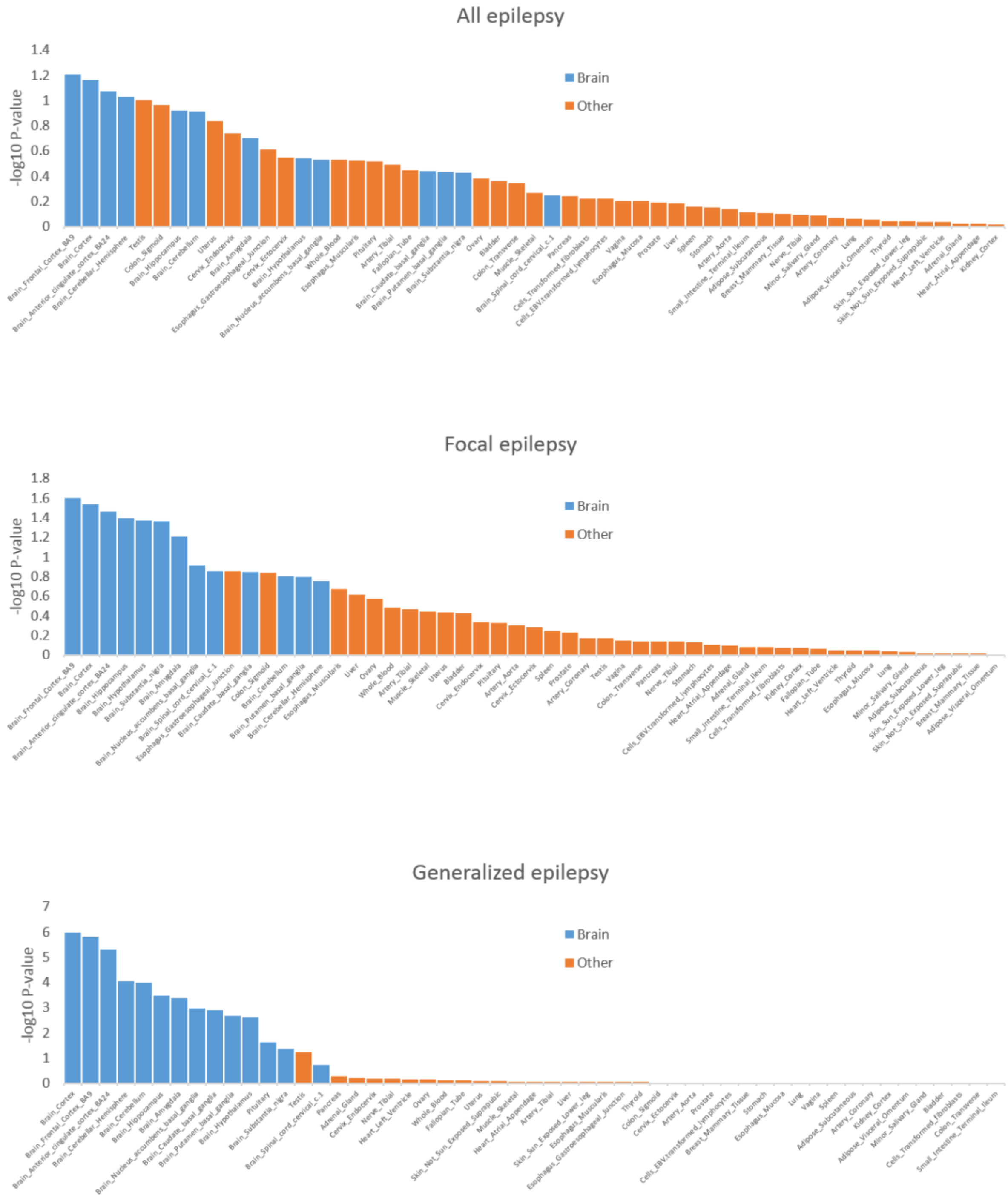

Supplementary Figure 12: MAGMA gene expression enrichment analysis *to test enrichment of gene expression in any of 53 tissues (from the GTEx consortium database) in (top) all epilepsy, (middle) focal epilepsy and (bottom) genetic generalized epilepsy GWAS.*

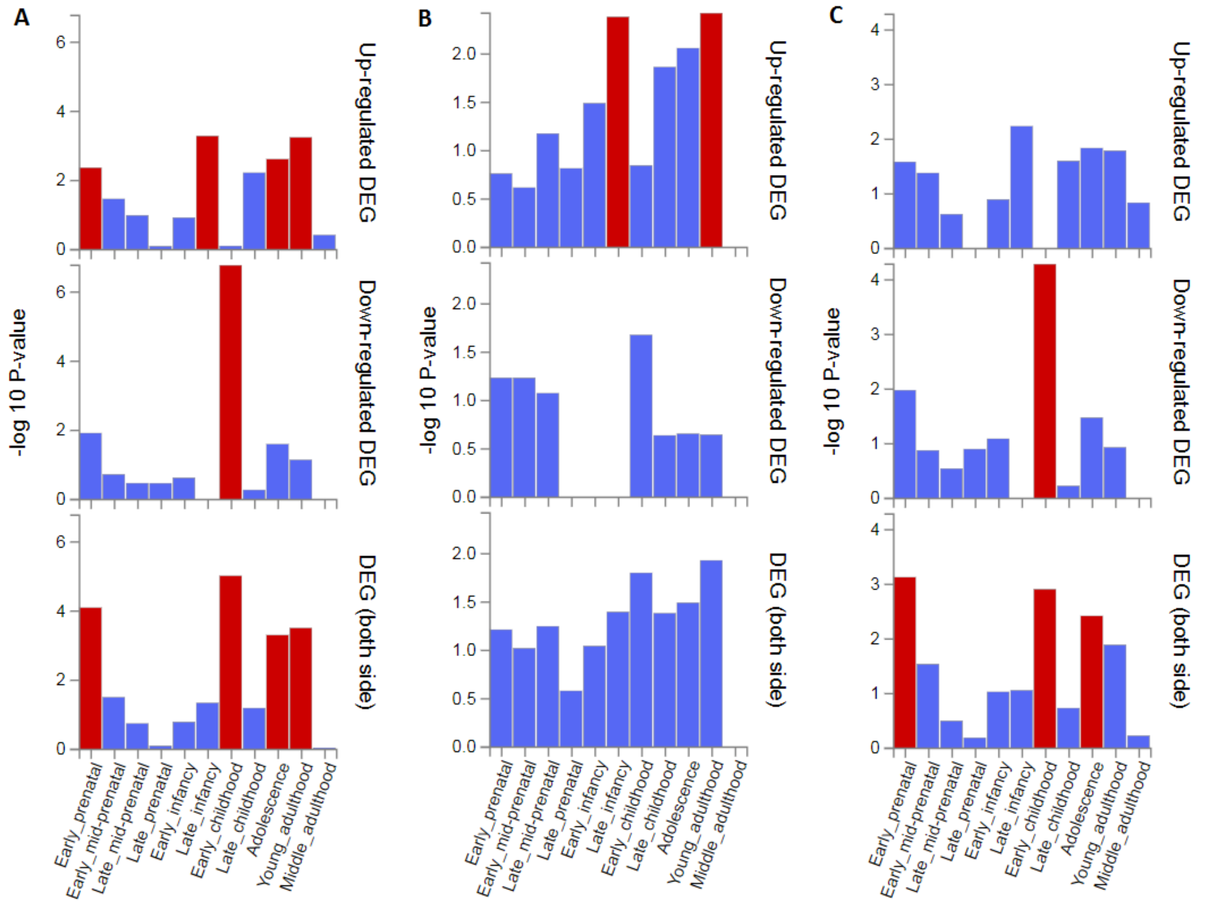

*Supplementary Figure 13: Differentially expressed genes across the lifespan for all (**A**), focal (**B**) and generalized epilepsies (**C**). Significantly enriched DEG sets (P_{bon} < 0.05) are highlighted in red. Gene transcription data from postmortem human brain specimens from the Brainspan Atlas of the Developing Human Brain was used.*

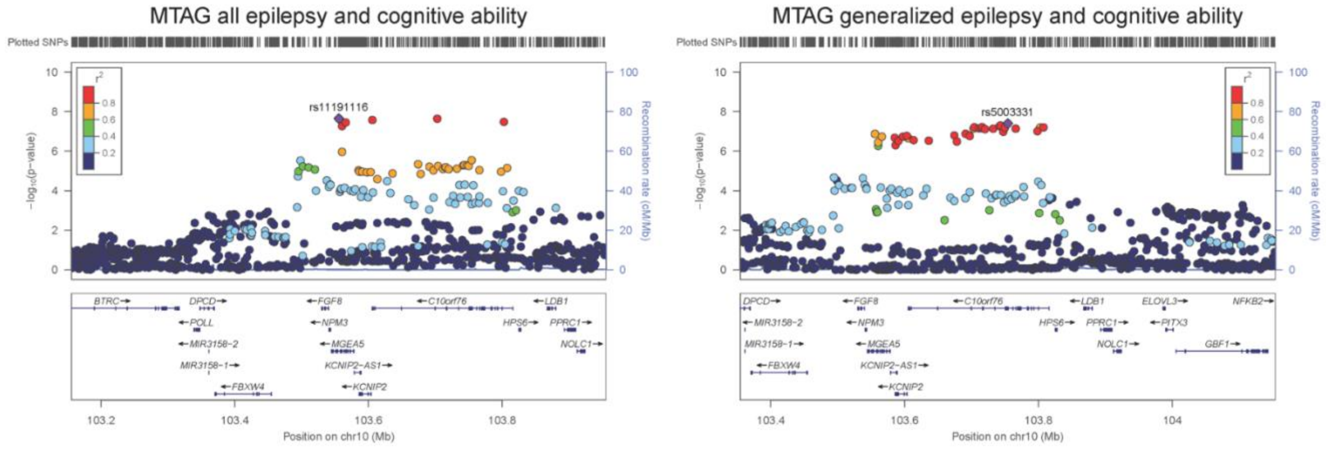

Supplementary Figure 14: Results of MTAG analysis *when all epilepsy (left) and genetic generalized epilepsy (right) GWAS are paired with the genetically correlated GWAS of cognitive ability.*

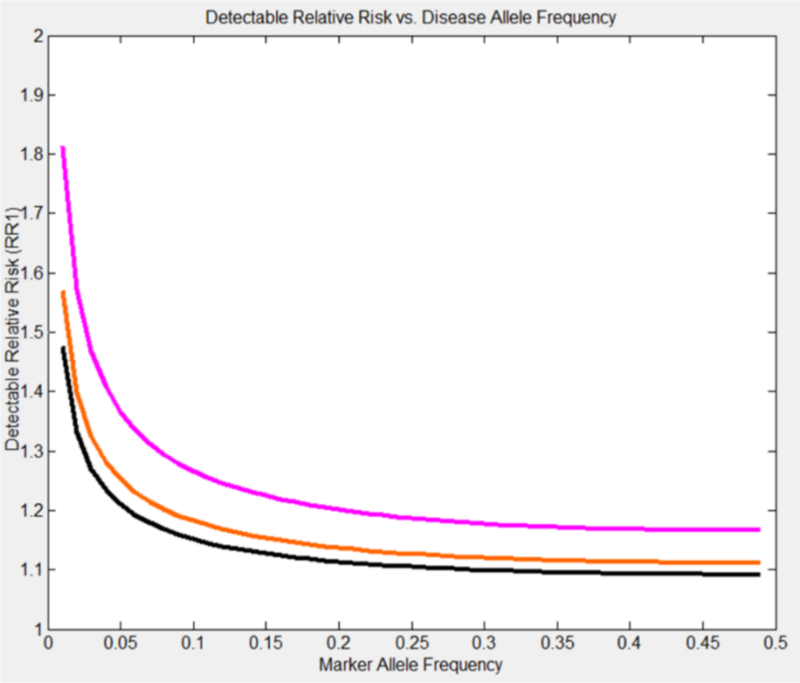

Supplementary Figure 15: Power curves for GWAS*. We estimated to have 80% power to detect a genetic association for all epilepsy (black), focal epilepsy (orange) and genetic generalized epilepsy (pink).*

Supplementary References

1. Meng, W. *et al.* A Genome-Wide Association Study Finds Genetic Associations with Broadly-Defined Headache in UK Biobank (N = 223,773). *EBioMedicine* **28**, 180-186 (2018).

2. Lambert, J.-C. Meta-Analysis of 74,046 Individuals Identifies 11 New Susceptibility Loci for Alzheimer's Disease. *Nat. Genet.* **45,** 1452–1458 (2013).

3. Simón-Sánchez, J. *et al.* Genome-wide association study reveals genetic risk underlying Parkinson's disease. *Nat. Genet.* **41,** 1308–1312 (2009).

4. NINDS Stroke Genetics Network (SiGN), International Stroke Genetics Consortium (ISGC). Loci associated with ischaemic stroke and its subtypes (SiGN): a genome-wide association study. *Lancet. Neurol.* **15,** 174–84 (2016).

5. van't Hof, F. N. G. *et al.* Shared genetic risk factors of intracranial, abdominal, and thoracic aneurysms. *J. Am. Heart Assoc.* **5** (2016).

6. Feenstra, B. *et al.* Common variants associated with general and MMR vaccine-related febrile seizures. *Nat. Genet.* **46,** 1274–1282 (2014).

7. Gormley, P. *et al*. Meta-analysis of 375,000 individuals identifies 38 susceptibility loci for migraine. *Nature genetics* 856–866 (2016).

8. Ripke, S. *et al.* Biological insights from 108 schizophrenia-associated genetic loci. *Nature* **511,** 421–427 (2014).

9. Sklar, P. *et al.* Large-scale genome-wide association analysis of bipolar disorder identifies a new susceptibility locus near ODZ4. *Nat. Genet.* **43,** 977–985 (2011).

10. Ripke, S. *et al.* A mega-analysis of genome-wide association studies for major depressive disorder. *Mol. Psychiatry* **18,** 497–511 (2013).

11. Middeldorp, C. M. *et al.* A Genome-Wide Association Meta-Analysis of Attention-Deficit/Hyperactivity Disorder Symptoms in Population-Based Paediatric Cohorts. *J. Am. Acad. Child Adolesc. Psychiatry* **55**, 896-905 (2016).

12. Bentham, J. *et al.* Genetic association analyses implicate aberrant regulation of innate and adaptive immunity genes in the pathogenesis of systemic lupus erythematosus. *Nat. Genet.* **47,** 1457–1464 (2015).

13. Sawcer, S. *et al.* Genetic risk and a primary role for cell-mediated immune mechanisms in multiple sclerosis. *Nature* **476,** 214–219 (2011).

14. Sniekers, S. *et al.* Genome-wide association meta-analysis of 78,308 individuals identifies new loci and genes influencing human intelligence. *Nat. Genet.* **49,** 1107–1112 (2017).

15. Van Den Berg, S. M. *et al.* Harmonization of neuroticism and extraversion phenotypes across inventories and cohorts in the Genetics of Personality Consortium: An application of item response theory. *Behav. Genet.* **44,** 295–313 (2014).

16. de Moor, M. H. M. *et al.* Meta-analysis of genome-wide association studies for personality. *Mol. Psychiatry* **17,** 337–349 (2012).
