# Supplementary material for: Genome-wide mega-analysis identifies 16 loci and highlights diverse biological mechanisms in the common epilepsies
Source: Nat Commun. 2018 Dec 10;9:5269. doi: 10.1038/s41467-018-07524-z (PMC6288131; doi:10.1038/s41467-018-07524-z)
Supplement: Supplementary file 3 — Description of Additional Supplementary Files [file 41467_2018_7524_MOESM3_ESM.pdf]

Description of Additional Supplementary Files

File Name: Supplementary Data 1
Description: Biological prioritization criteria for all 146 genes that are mapped to genome-wide significant loci from all analyses. Biological prioritization criteria satisfied are displayed with filled red boxes. The total score denotes the number of criteria satisfied. TWAS: significant TWAS association (based on data from the CommonMind Consortium); eQTL: significant eQTL within locus (based on data from the ROS/MAP projects); brain expression: the gene is preferentially expressed in the brain; missense in gene: epilepsy GWAS missense variant in locus; PPI: gene prioritized by protein-protein interaction; KO mouse: relevant knockout mouse phenotype.

File Name: Supplementary Data 2
Description: List of drugs (n=166) that target biological prioritized genes from our GWAS. Data was obtained from the Drug-Gene interaction database (http://dgidb.org).
